# A Retrospective Study of Ultrasound Characteristics and Macroscopic Findings in Confirmed Malignant Pleural Effusion

**DOI:** 10.1155/2019/5628267

**Published:** 2019-02-18

**Authors:** Nevenka Piskac Zivkovic, Igor Cikara, Nina Petra Novak, Boris Brkljacic, Neven Tudoric

**Affiliations:** ^1^University Hospital Dubrava, Zagreb, Croatia; ^2^Faculty of Medicine Osijek, University Josip Juraj Strossmayer Osijek, Croatia; ^3^School of Medicine, University of Zagreb, Zagreb, Croatia

## Abstract

**Background:**

A definitive diagnosis of malignant pleural effusion (MPE) is reached by cytological or histological assessment, but thorough analysis of the ultrasound features of the effusion as well as pleural thickening or nodularity can also be of significant diagnostic help.

**Objective:**

To assess the relationship of specific ultrasound characterisctics and macroscopic features of confirmed malignant pleural effusion, thus increasing the diagnostic potential of thoracic ultrasound.

**Methods:**

The findings of thoracic ultrasonography performed prior to initial thoracentesis in 104 patients with subsequently confirmed malignant pleural effusion were analyzed with regard to the macroscopic features of the pleural effusion.

**Results:**

Distribution in terms of frequency of hemorrhagic/sanguinolent (n=64) in relation to nonhemorrhagic transparent/opaque (n=40) MPE, regardless of their ultrasound characteristics, did not yield a statistically significant correlation (p=0.159). Conversely, the frequency distribution of hemorrhagic pleural effusions (n=8) in relation to nonhemorrhagic effusions (n=1), in the group of septated MPE, showed a statistically significant difference (p<0.001). The least number of patients (0.96%) had a complex septated MPE combined with the macroscopic appearance of a serous/transparent nonhemorrhagic effusion, which suggests that this combination is a sporadic occurrence and may have a diagnostic significance for this patient group.

**Conclusion:**

The incidence of specific combinations of the ultrasound characteristics and macroscopic appearance of MPEs showed different frequency distributions, which may improve the diagnostic value of thoracic ultrasound in this patient population.

## 1. Introduction

Pleural effusion is a common manifestation of various malignancies, suggesting advanced disease and a poor prognosis. Approximately 30% of malignant pleural effusions originate from lung carcinoma and result in survival rates of 8-10 months [1]. Detection of pleural effusion often leads to prompt implementation of standardized diagnostic procedures with thoracocentesis as the initial step.

Thoracic ultrasound (TUS) is an important, often initial, diagnostic method for the detection and localization of pleural effusion, as well as for the safe performance of further invasive diagnostic procedures. Since it enables real-time visualization, TUS significantly increases diagnostic accuracy, considerably diminishing the number of potential complications. A detailed thoracic ultrasound examination incorporates the analysis of sonographic features of the effusion, the visceral and parietal pleura, and the visible lung parenchyma. Although the definitive diagnosis of malignant effusion is made from a cytological or histological assessment, a thorough analysis of the ultrasound findings has significant diagnostic value.

According to Yang et al. [2], pleural effusion is classified as anechoic, complex septated, complex nonseptated, or homogeneously echogenic. The echogenicity of the pleural effusion is assessed by comparing it with the echogenicity of the liver (hypoechoic, isoechoic, and hyperechoic), while the reference value for anechogenicity is the echogenicity of bile in the gallbladder. The terms “complex” or “heterogeneous” are used to denote findings of echogenic zones within an anechoic effusion. Fibrinous septation is a relatively common finding in pleural effusion and varies in intensity, ranging from a few separated, often floating, fibrin strands to dense reticular structures with a honeycomb appearance [3–5]. Fibrinous septation is the consequence of an increased amount of proteins in the effusion, therefore being a common finding in exudates, including tuberculous, pleural empyema, hematothorax, and parapneumonic effusions [6, 7].

According to Yang et al. [2] transudate pleural effusion is always anechoic, whereas exudates, both malignant and nonmalignant, may be anechoic or echogenic. The authors reported findings of anechoic pleural effusion in 27% of nonmalignant and 40% of malignant pleural effusions, a similar distribution of various types of echogenic effusions. Conversely, Bugalho et al. [7] found only 5% of anechoic malignant effusions, which is in line with the results of others [6, 8]. In most cases, the malignant effusion presented features of complex nonseptated effusion [2].

The potential cause for the lower incidence of fibrinous septation in malignant effusion has been analyzed at the molecular level. It was proposed to be the consequence of increased fibrinolytic activity in malignant effusion resulting from a higher level of tissue plasminogen activator (tPA). In contrast, tuberculous exudates were characterized by an increased level of the inhibitor type-1 of tissue plasminogen activator (PAI -1) and tumor necrosis factor alpha (TNF-alpha) [9, 10]. The fibrinous septation was also reported to be a consequence of repeated thoracocenteses and pleurodesis, where increased levels of inflammatory cytokines (TNF-alpha, IL-1, IL-5, IL-6, and IL-8) were found [11, 12].

Malignant pleural effusion has biochemical features of exudate and only rarely presents as transudate [13, 14]. Macroscopically, malignant pleural effusions can be serous, sanguinolent, or hemorrhagic. Cytological analysis reveals predominance of lymphocytes, macrophages, and mesothelial cells, whereas there are usually less than 25% of polymorphonuclears and between 8 and 12% of eosinophils [15] found.

A complete chest sonography includes an estimate of pleural thickness, possible detection of pleural nodes, and an examination of the adjacent lung parenchyma (presence of the air bronchogram or possible pulmonary consolidation). TUS also enables measurement of the thickness of the diaphragm, as well as the possible detection of liver metastases. Although the finding of the thickened visceral, parietal, and diaphragmal pleura is common in malignant effusions, if it is less than 1 cm, it does not have specific diagnostic relevance [7, 8]. On the contrary, pleural thickening greater than 1 cm, along with the finding of pleural nodes, is very indicative. Bugalho et al. [7] reported these findings in 79% of malignant and only 9% of nonmalignant pleural effusions. The presence of air bronchogram is more common in nonmalignant effusions.

As proof of malignant etiology, cytological analysis of the pleural effusion sampled at initial thoracocentesis has a sensitivity of 62%. The repeated procedures increase the sensitivity up to 72% (49-91%) [16]. This indicates that for a large number of patients further diagnostic evaluation is necessary. Targeted TUS-guided pleural biopsy increases the number of successfully diagnosed malignant effusions with sensitivity of almost 90% [17–19]. However, in the case of some pleural effusions collected in patients with lung carcinoma, the presence of malignant cells or pleural involvement cannot be detected. Such cases are classified as paramalignant pleural effusions resulting from diverse causes, including atelectasis, pulmonary embolism, hypoalbuminemia, chylothorax, radiotherapy, and chemotherapy [20].

The objective of our study was to assess the relationship of specific ultrasound characterisctics and macroscopic features of confirmed malignant pleural effusion, thus increasing the diagnostic potential of thoracic ultrasound.

## 2. Materials and Methods

This retrospective, single-center study was conducted at the Division of Pulmonology of the University Hospital Dubrava in Zagreb, Croatia, during a 4-year period (January 2014–June 2018). It has been approved by the institutional Ethics Committee.

### 2.1. Subjects

The studied subjects were selected among patients referred for thoracic ultrasonography because of suspected pleural effusion at the standard chest X-ray. In the studied group of patients, the malignant etiology of effusion was subsequently established using regular diagnostic procedures. This means that, in case of repeatedly negative cytoanalysis of effusion, ultrasound- or CT-guided pleural needle biopsy was performed. A few patients underwent video-assisted thoracoscopy (VATS) with biopsy of the pleura and/or lung parenchyma. All collected samples were analyzed at the Department of Cytology and Pathology in the same institution. Patients with contraindications for thoracentesis and those who did not give their consent to participate in the study were excluded.

### 2.2. Ultrasonography

The procedure was performed using the Aloka 7 Utrasound System (Aloka, Japan). A 2-5 MHz convex probe was used for the analysis of the pleural effusion, visceral pleura, and lung parenchyma, whereas the parietal pleura and thoracic wall were examined with the use of a linear 5-10 MHz probe. The examination was conducted with the patient placed in the sitting position, by a pulmonologist experienced in the use of TUS (15Y). The thoracic cavity was systematically examined by moving the probe from dorsal to ventral along the intercostal spaces from the diaphragm to the lung apices.

Based on the ultrasound features, the pleural effusions were classified as anechoic, homogeneously echogenic, complex septated, or complex nonseptated. Furthermore, the appearance and thickness of the parietal, visceral, and diaphragmal pleura were analyzed, as well as the available lung parenchyma and the thoracic wall structures. After ultrasonography, ultrasound-guided pleural puncture was performed in accordance with the British Thoracic Society (BTS) guidelines [20]. The gross features of the obtained samples (transparent clear to pale yellow, pale yellow with cloudy appearance, opaque, sanguinolent, and hemorrhagic) were recorded, before the samples were referred for further biochemical, microbiological, and cytologocal assessment.

### 2.3. Statistical Analysis

The collected data were entered into the Excel spreadsheet (MS Office 2007) and the cumulative frequency for the ultrasound features and gross findings of the malignant pleural effusions was calculated. Statistical analysis of the frequency table was done by Chi-square test, using the SAS Studio 3.5 statistical software package (SAS Institute Inc., Cary, NC, USA). P<0.05 was considered statistically significant.

## 3. Results

In the above-mentioned 4-year period, 104 patients (55 females, 49 males, mean age 69 years) with confirmed MPE were recorded by means of retrospective analysis. The malignant nature of the effusion was confirmed by the finding of malignant cells in pleural effusion samples from 72 (69%) patients, in transthoracic pleural biopsy samples from 26 (25%) patients, and in samples obtained by surgical pleural biopsy during VATS from 6 (6%) patients. In 97 patients the pleural effusion was a result of solid tumor metastasis, while 7 patients had a dissemination of hematologic disease. In the majority of the cases, the primary sites of the tumors were the lungs (41%), organs of the gastrointestinal tract (24%), and the breasts (13%).  Table 1 lists the primary sites as well as the types of tumors and hematologic diseases diagnosed in the patients with MPEs.

**Figure 1:**
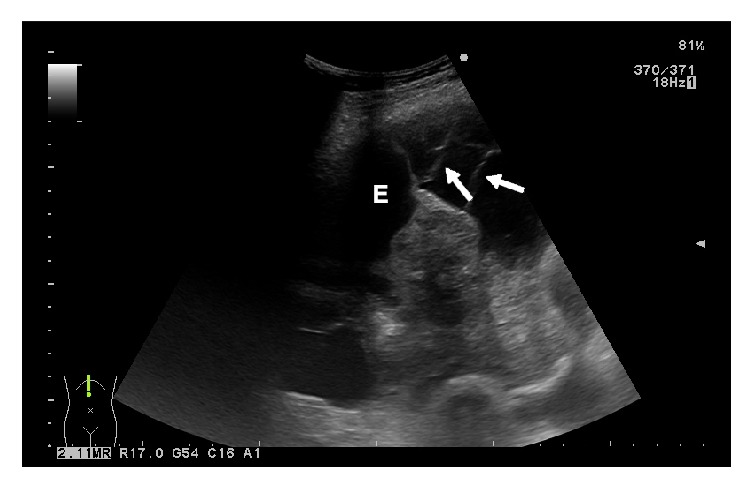
Typical ultrasonographic image of malignant pleural effusion with fibrinous septation. Fibrin strands within a pleural effusion can be seen (white arrows); E = pleural effusion.

**Figure 2:**
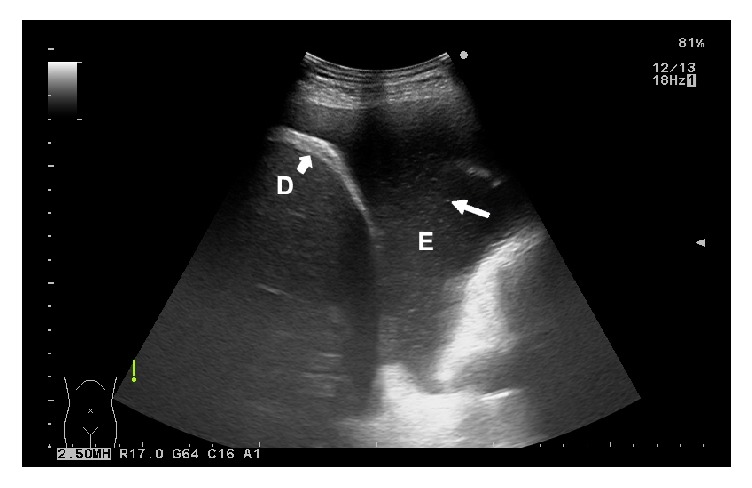
Typical ultrasonographic image of complex nonseptated malignant pleural effusion. Heterogeneously hyperechoic spots inside effusion (right white arrow) without fibrinous septations can be seen. D = diaphragm (left bold white arrow); E = pleural effusion.

Based on the ultrasound features, 95 (91.35%) patients had a nonseptated pleural effusion (in 11 patients the pleural effusion was classified as anechoic, in 16 patients as homogeneously echogenic, and in 68 patients as complex nonseptated), and 9 (8.65%) patients were found to have a septated pleural effusion.  Table 2 lists the ultrasound characteristics of the MPEs as well as their gross features.  Figure 1 shows the typical ultrasound finding of fibrinous septations in a complex septated pleural effusion, and in Figure 2 a complex nonseptated pleural effusion can be seen.

According to the macroscopic characteristics, in 64 patients (62%) pleural effusion was sanguinolent/hemorrhagic, in 15 patients (14%) transparent and clear, while in the remaining 25 patients (24%) the effusion was pale yellow with cloudy appearance or opaque. The frequency distribution of the hemorrhagic/sanguinolent (n=64) in comparison with the nonhemorrhagic transparent or cloudy/opaque MPEs (n=40), regardless of their ultrasound characteristics, did not yield a statistically significant correlation (p=0.159).

The frequency distribution of the septated (n=8) in relation to the nonseptated pleural effusions (n=56) within the group of hemorrhagic effusions showed a statistically significant difference (p=0.0001).

In view of the specific ultrasound findings, special attention was given to the analysis of pleural effusions with fibrinous septations. Out of the 9 patients with MPEs with fibrinous septations, 3 had metastasis originating from lung tumors, in 2 cases the primary malignancies were adenocarcinomas of the lung, and there was one case of small cell lung carcinoma. Out of the remaining 6 complex septated pleural effusions, three were caused by the propagation of solid extrathoracic tumors (gastric carcinoma, melanoma, and papillary urinary bladder carcinoma), and 3 were the result of malignant hematologic disease dissemination (non-Hodgkin lymphoma and acute myelogenous leukemia). In 8 patients from this group the pleural effusion was sanguinolent or hemorrhagic, and only one patient (non-Hodgkin lymphoma) had a transparent serous pleural effusion. The frequency distribution of hemorrhagic pleural effusions (n=8) as compared to nonhemorrhagic effusions (n=1), within the group of MPEs with fibrinous septations, showed a statistically significant difference (p<0.0001). From the data listed in Table 2 it can be concluded that the combination of a macroscopically nonhemorrhagic pleural effusion and fibrinous septations was a sporadic occurrence in the MPE group, found only in one of the 104 patients.

Pleural nodularity was established in 53 patient samples, 75% of which were macroscopically hemorrhagic/sanguinolent. Pleural thickening (>10 mm) was confirmed in 45 patients, 64% of which had gross features of hemorrhagic/sanguinolent pleural effusion.

The majority of the analyzed pleural effusions were lymphocytic (>50% lymphocytes). The number of polymorphonuclears was lower than 25%, and the amount of eosinophils was less than 12%. In 3 (3%) patients the malignant pleural effusion had transudate characteristics.

## 4. Discussion

This is the first study to compare the macroscopic characteristics with the ultrasound findings in MPE. Consistent with the results of other authors [21, 22], distribution analysis of the macroscopic findings in MPE, regardless of the ultrasound features, did not yield a statistically significant difference (p=0.159). Yang et al. [2] were the first to describe the ultrasound characteristics of MPE, whereas Qureshi et al. [8] repoted that malignancies had been diagnosed by TUS in 26/33 patients. Out of 7 false-negative results, the CT findings were negative in 6 (86%) patients. The authors concluded that ultrasound findings of pleural nodularity accompanied by a thickening of the parietal costal pleura by >10 mm and the diaphragmatic pleura by >7 mm represented a significant predictor of a malignant etiology of the pleural effusion, with a procedure sensitivity of 73% and a specificity of 100%. It has to be emphasized that pleural nodularity was confrmed in specific pleural effusions as well [7]. Due to that fact, the finding of pleural nodularity is not considered pathognomonic for MPE, especially in regions where tuberculosis is common, although it still remains a significant predictor of malignancy in undiagnosed pleural effusion (sensitivity 78.8%, specificity 91.0%). In our patient cohort pleural nodularity was established in 51% of the cases. In 75% of these patients the effusion was hemorrhagic or sanguinolent. Consistent with that, a >10 mm thickening of the parietal pleura was confirmed in 43% of the patients, out of which 56% had a hemorrhagic or sanguinolent effusion. Furthermore, the literature describes two more ultrasound findings, i.e., the invasion of thoracic wall structures and the presence of liver metastasis, which suggest a malignant etiology of the pleural effusion with a high sensitivity and specificity [7, 23]. These ultrasound criteria are present in a smaller number of MPE patents. Negative cytological findings combined with ultrasound criteria which suggest a malignant etiology of the pleural effusion require a further diagnostic workup and obtaining samples for pathohistological analysis.

**Figure 3:**
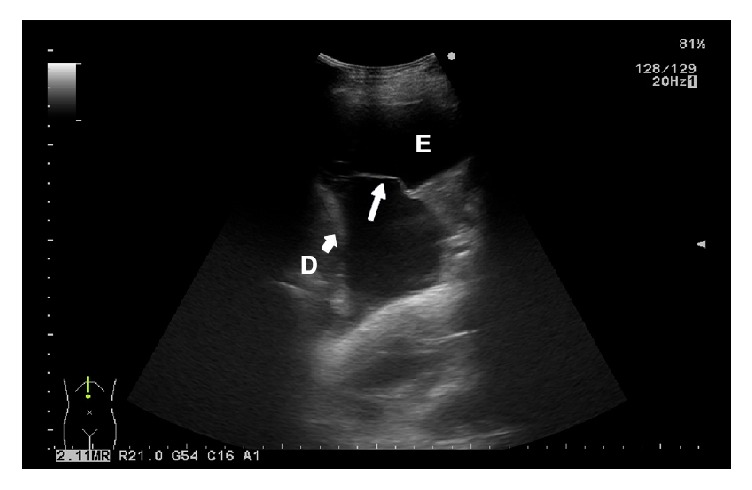
Floating visceral pleura due to atelectasis of the lung lobe (right white arrow). D = diaphragm (left bold white arrow); E = pleural effusion.

**Figure 4:**
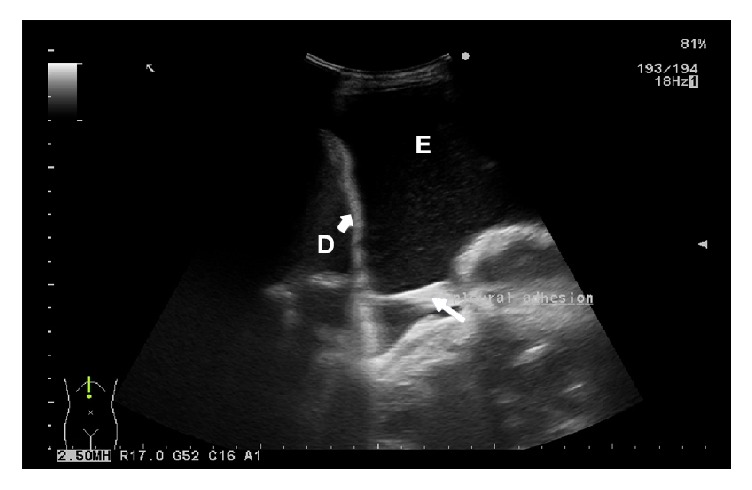
Pleural adhesions. Pleural adhesions (right white arrow) with sharp contours, hyperechoic, connecting the diaphragmatic pleura and parenchyma of the collapsed lung. D = diaphragm (left bold white arrow); E = pleural effusion.

Two further ultrasound characteristics with a higher incidence in MPE in relation to nonmalignant pleural effusion (NMPE) are the absence of air bronchograms in the peripheral lung infiltrate (p=0.001) and the absence of fibrinous septations in the pleural effusion (p=0.006) [7]. The cited parameters display a high sensitivity, but low specificity. The absence of air bronchograms has a sensitivity of 92.4% and a specificity of 31.3% [7]. False-negative findings are most often due to malignant obstructive pneumonia. Fibrinous sepations are a relatively rare finding in MPE, reported by various authors at an incidence of 4-7.5% [6–8]. TUS is a very reliable method for the assessment of septations in pleural effusion, with a sensitivity of 92.4% [7]. However, due to its low specificity (25%), the analysis of fibrinous septations has a limited diagnostic value. The possibility of errors in the ultrasound evaluation of fibrinous septations in the effusion needs to be taken into account. This applies to the findings of floating visceral pleura of an atelectatic lung lobe (Figure 3) and pleural adhesions (Figure 4), which are common in effusions with transudate features that have been present for a longer period of time. They are usually supradiaphragmatic, with cytological features of reactively changed, macrophage-transformed mesothelial cells [24].

Taking into account the results of studies conducted so far, and with the aim of improving the effectiveness of TUS in MPE patients, macroscopic findings of pleural effusion were analyzed retrospectively within the context of their ultrasound characteristics. Upon statistical analysis and the correlation of frequency distributions of specific combinations of macroscopic appearance and ultrasound characteristics of MPEs, significant results were obtained. The frequency distribution of hemorrhagic/sanguinolent (n=64) in relation to nonhemorrhagic transparent/opaque (n=40) malignant pleural effusions, regardless of their ultrasound characteristics, did not yield a statistically significant correlation (p=0.159). In contrast, the frequency distribution of hemorrhagic (n=8) as compared to nonhemorrhagic pleural effusions (n=1), within the group of malignant pleural effusions with fibrinous septations, showed a statistically significant difference (p<0.0001). The least number of patients (0.96%) had a complex septated MPE combined with the macroscopic appearance of a serous/transparent nonhemorrhagic effusion, which suggests that this combination is a sporadic occurrence and may have a diagnostic significance for this patient group.

The authors are aware of the study limitations, particularly in view of the fact that it was a retrospective investigation which did not include patients with nonmalignant pleural effusion. We believe that prospective study, which would also include patients with nonmalignant pleural effusion, would help in better evaluation of diagnostic value of thorasis ultrasound in the assessment of malignant etiology in undiagnosed pleural effusions.

## Figures and Tables

**Table 1:** Primary origin and type of malignant disease in patients with malignant pleural effusion.

Site and type of tumor	Patients (N)
Lung adenocarcinoma	34

Small cell lung carcinoma	5

Non-small cell lung carcinoma	4

Pleural mesothelioma	6

Gastric adenocarcinoma	9

Colon adenocarcinoma	16

Invasive ductal breast cancer	14

Ovarian carcinoma	4

Endometrial carcinoma	2

Thymic carcinoma	1

Papillary carcinoma of the urinary bladder	1

Melanoma	2

Non-Hodgkin lymphoma	5

Acute myeloid leukemia	1

**Table 2:** Ultrasound features and macroscopic appearance of malignant pleural effusions.

Ultrasound characteristic					Macroscopic finding					
					Sanguinolent/ hemorrhagic		Serous transparent		Serous opaque	
	n	%	n	%	n	%	n	%	n	%
Complex septated	9	8.65	9	8.65	8	88.89	1	11.11	0	0.00

Homogeneously echogenic	16	15.39	95	91.35	56	58.95	14	14.74		
Anechoic	11	10.58							25	26.31
Complex non-septated	68	65.38								

Total	104	100.00	104	100.00						

## Data Availability

The data used to support the findings of this study are available from the corresponding author upon request.
